# *Plasmodium falciparum* parasitaemia and malaria among pregnant women at first clinic visit in the mount Cameroon Area

**DOI:** 10.1186/s12879-015-1211-6

**Published:** 2015-10-22

**Authors:** Judith K. Anchang-Kimbi, Vera Ngenwie Nkweti, Helen Ngum Ntonifor, Tobias O. Apinjoh, Rolland Bantar Tata, Hanesh Fru Chi, Eric Akum Achidi

**Affiliations:** Department of Zoology and Animal Physiology, University of Buea, Buea, 63, Cameroon; Department of Biological Sciences, University of Bamenda, Bamenda, Cameroon; Department of Biochemistry and Molecular Biology, University of Buea, Buea, 63, Cameroon; Department of Molecular Parasitology, University of Buea, Buea, 63, Cameroon

**Keywords:** *P. falciparum* infection, Malaria, Pregnancy

## Abstract

**Background:**

Pregnant women in malaria endemic areas are at high risk of *P. falciparum* infection and its complications. This study investigated the prevalence and risk factors for *P. falciparum* infection and malaria among pregnant women reporting for first antenatal care (ANC) clinic visit in the mount Cameroon area.

**Methods:**

Venous blood samples from consented pregnant women were screened for malaria parasitaemia by light microscopy. Haemoglobin levels, white blood cell (WBC) counts, lymphocyte counts and percentage were determined using an automated haematology analyser. Socio-demographic/economic data, environmental factors and use of malaria control measures were documented. Univariate and multivariate statistical analyses were used.

**Results:**

Sixty-eight (22.4 %; N = 303) of the women enrolled were positive for *P. falciparum* parasitaemia. Malaria parasitaemia was significantly (P < 0.001) associated with febrile illness. The overall prevalence of malaria and asymptomatic infection was 16.0 % (95 % CI = 11-20 %) and 10.5 % (95 % CI = 7.3-15 %) respectively. A greater proportion of the malaria cases (61 %) reported at the clinic during unscheduled days meanwhile women with asymptomatic parasitaemia mostly (92.8 %) seek for ANC during scheduled clinic days. Lower lymphocyte percentage was significantly associated with increase parasite density (r = − 0.34; P = 0.011) and febrile status (MU = 2.46; P = 0.014). While age and gravidity were significant factors associated with *P. falciparum* infection and/or malaria, the presence of bush and/or standing water around human residence was an independent risk factor of *P. falciparum* parasitaemia (OR = 3.3: 95 % CI = 1.6 – 7.0; P = 0.002) and malaria ( OR = 5.2: 95 % CI = 2.0 – 14; P = 0.001). Being unmarried was significantly associated with increase risk (OR = 2.6:95 % CI = 1.1 – 6.0; P = 0.032) of *P. falciparum* parasitaemia. Similarly, single women (938) had a significantly higher (t = 2.70; P = 0.009) geometric mean parasite density (GMPD) compared with married women (338).

**Conclusion:**

Marital status and human residence in areas with bushes and/or standing water modify risk of *P. falciparum* infection and malaria. Education on early ANC attendance and environmental sanitation are important public health targets for malaria control in pregnancy in this setting.

## Background

Pregnant women in malaria endemic areas are at high risk of *P. falciparum* infection and its complications which are severe and multiple [1]. These may include placental malaria, fever, maternal anaemia, foetal parasite exposure, congenital infection, low birth weight (LBW) (due to preterm delivery (PTD) and intrauterine growth retardation (IUGR)), infant mortality and anaemia [1]. It is generally assumed that due to acquisition of partial immunity to malaria, parasitaemic women living in areas of stable transmission are rarely symptomatic [2]. However, various studies show that in stable malaria endemic regions, pregnant women have frequent episodes of malaria from early to mid pregnancy [3–5]. It is recommended that pregnant women should be given antimalarial drugs at their first antenatal visit and perhaps earlier in pregnancy whether or not they show symptoms to prevent adverse effect of malaria on the mother and foetus [6]. In areas of stable malaria transmission of sub-Saharan Africa, intermittent preventive treatment in pregnancy (IPTp) with sulfadoxine-pyrimethamine (SP) (IPTp-SP) is recommended for all pregnant women at each scheduled antenatal care visit (at least one month apart) up to the time of delivery [7]. Treatment of falciparum malaria involves administration of quinine plus clindamycin (if available) and if treatment fails, Artesunate combined therapy (ACT) is recommended [6, 8].

Longitudinal cohort studies investigating the clinical presentation of malaria parasite infection in pregnant women showed that during routine ANC (scheduled) visits, malaria is less common [4, 9, 10]. However, studies in Mozambique [4] and Benin [5] revealed that during unscheduled visits, about 90 % of pregnant women infected with malaria parasite had symptoms. Symptoms commonly associated with malaria parasitaemia include fever, headache and shivering [5]. It is possible that in malaria stable transmission areas, the burden of malaria is been underestimated, as most studies have been carried out in women reporting for scheduled visits or at delivery to investigate placental malaria [6, 9, 10].

Clinical manifestations of *P.falciparum* infection may largely depend on the intensity and stability of malaria transmission in the local environment [11]. Other factors commonly reported to increase risk of malaria among pregnant women include; younger age, primigravidity, second and third trimester of gestation [3, 10], level of education [10, 12] and rainy season [3]. Malaria alters haematological parameters with low platelet, WBCs and lymphocyte counts being the most important predictors of malaria infection. Furthermore, when used in combination with other clinical and microscopy methods, these parameters could improve malaria diagnosis and treatment in people living in malaria endemic areas [13, 14]. Most recent studies have reported haematological changes in malaria in children and adults [13–15]. Malaria-related haematotological alterations in pregnant women warrant investigation.

Previous findings in the mount Cameroon area show that fever history is common among pregnant women and this usually correlates with the time of first clinic visit [16, 17]. Pregnant women in this area may experience malarial fever episodes before their first ANC clinic visit. Thus, this study investigated the prevalence of *P. falciparum* infection and malaria among pregnant women reporting for first ANC clinic visit during scheduled and unscheduled clinic days at some health facilities in the study area. Secondly, the study assessed demographic, socio-economic, environmental, haematological factors to identify risk factors associated with *P. falciparum* infection and malaria in pregnancy in this setting.

## Methods

### Study area

This study was carried out in Mutengene and Muea Integrated Medical Centres located in the Mt. Cameroon Area, Fako Division, South West Region. These medical centres are government-owned institutions that offer antenatal care, preventive and curative services at affordable costs for the middle and low- income population. Both health facilities are highly accessible, facilitating utilisation of ANC services.

The Mt. Cameroon Area has an equatorial climate made up of a long rainy season which runs from March to October with maximum rainfall usually recorded in the months of August and September. The dry season runs from November to February. Malaria parasite transmission is perennial and *P. falciparium* accounts for up to 96 % of malaria parasite infections in this area [18]. Malaria is meso-endemic during the dry season but becomes hyper-endemic in the rainy season, with incidence peaking in July to October [18]. Entomological surveys carried out in the study area show that the anopheline mosquitoes are predominant and diverse: *Anopheles gambiae* s.*l*. (56.86 %), *An. funestus s.l.* (32.57 %), *An. hancocki* (9.38 %) and *An. nili* (1.18 %). All anopheline species are highly anthropophilic and exophagic (exceedingly for *An. gambiae*) with a human blood index (HBI) of 99.05 % [19]. *Anopheles gambiae* is proportionately more abundant throughout the year and show peak of abundance towards the rainy season. Equally, the high survival rates of the malaria vectors (mean probability of daily survival of 0.92, annual mean life expectancy of 21.9 days and expected mean infective life of 7.4 days) suggest a high vector potential for the species [19]. The overall vector infectivity rate is estimated at 3.93 infective bites/person/night [20]. In the Mt Cameroon area, climate suitability zone for *An. gambiae s.s* is defined as total annual precipitation of 230–9224 mm, maximum annual temperature range of 25–31 °C and minimum annual temperature range of 18–20 °C. *An. funestus* thrives in zones with a total annual precipitation of 230–2817 mm, maximum annual temperature of 25–29 °C and minimum annual temperature of 20–23 °C. The mean maximum annual relative humidity is 88 % [19].

Mutengene is a semi-urban, road junction town located at about 220 m above sea level with a highly heterogonous population of approximately 40,000 inhabitants comprising people from most ethnic communities in Cameroon, some parts of neighbouring Nigeria, Niger and Ghana in search of fertile farmland and business opportunities [21]. Mutengene is characterised by mean temperature of 25.08 °C and mean relative humidity of 83.1 % (Cameroon Development Corporation (CDC) weather records, 2010).

Muea is a semi-rural setting located at an altitude of 562 m above sea level on the Eastern flank of the active volcanic Mt Cameroon with relative humidity of above 80 %, temperature range of 18–28 °C and an annual rainfall of about 4096 mm. Muea has a heterogeneous and multiethnic population of approximately 9,000 inhabitants, majority of whom are farmers [22].

### Ethics statement

Ethical clearance (No2013/0107/UB/FHS/IRB) was obtained from the University of Buea, Faculty of Health Sciences Institutional Review Board and administrative authorisation from the South West Regional Delegation of Public Health, Buea. Written informed consent was obtained before enrolment into the study.

### Study population

Pregnant women who reported for first ANC clinic visit on scheduled or unscheduled clinic days at the Mutengene or Muea Medical Centres and who gave their consent to participate in the study were enrolled. Pregnant women included those with or without fever.

### Study design

This prospective cross sectional study was carried out from March to August 2013 which corresponds to the period of intense malaria transmission in the mount Cameroon area. In the study area, routine ANC clinic visits (scheduled) are on Mondays and Thursdays while Tuesdays, Wednesday and Fridays are unscheduled days. Women reporting for first ANC clinic visit on scheduled or unscheduled clinic days and who volunteered to participate in the study were enrolled consecutively. At first scheduled or unscheduled visit, a structured questionnaire was used to document demographic information (age, residence, marital status), gynaecologic/obstetric history (parity status, gestational age (GA), pregnancy complications, trimester of first visit), socio-economic indicators (educational level, occupation, monthly income and knowledge of malaria in pregnancy (MiP) and environmental factors (house type, presence of bushy surroundings and/or standing water). Monthly income recorded was self reported. IPTp-SP/dosage and ITN usage were recorded. Temperature was recorded using a digital thermometer and fever was defined as temperature > 37.5 °C. Maternal peripheral venous blood (2 ml) was collected by venipuncture into EDTA tubes for haematological assessment and malaria parasite determination. Human immunodeficiency virus (HIV) infection status was determined for mothers enrolled. Women in this setting are offered free routine confidential HIV testing and counselling at first ANC clinic visit. All samples were transported on ice bath to the Malaria Research Laboratory, University of Buea for analysis.

### Laboratory analyses

#### Parasitological analysis

Thick and thin blood films were prepared, stained with 5 % Giemsa for 30 minutes [23] and examined under the x100 (oil immersion) objective of a UNICO® light microscope for the identification of the malaria parasite. Slides were declared negative if no asexual parasites or gametocytes were found after examining 100 high-power fields. For each of the positive slides, parasite density per μl of blood was determined in thick smear on the basis of the number of parasites per 200 leucocytes with reference to participants’ absolute WBC counts [23].

#### Haematological assessment

A complete blood count was ran using a Beckman coulter counter® (URIT 3000) following the manufacturer’s instructions, that automatically generated values for haemoglobin (Hb) levels, WBCs, lymphocytes and platelets. Anaemia was defined as Hb levels < 11.0 g/dl [24].

### Definitions and statistical analysis

Malaria (symptomatic infection) was defined as fever associated with *P. falciparum* parasitaemia while asymptomatic infection was defined as malaria parasitaemia with temperature of ≤ 37.5 °C. All data collected were entered into SPSS (Statistical Package for the Social Sciences) version 19 (SPSS, Inc, Chicago, IL, USA) for analyses. Malaria parasite densities were log transformed before analysis. Associations between *P. falciparum* parasitaemia and malaria with type of clinic visit, house surrounding, educational level, age, gravity, trimester of pregnancy, insecticide treated nets (ITNs) usage, IPTp-SP uptake and haematological parameters were evaluated using Pearson Chi-Square (χ^2^) test. Differences in group means were compared using ANOVA, Student’s-t-test, Mann Whitney U or Kruskall Wahlis test. Multinomial logistic regression model was used to determine risk factors associated with *P. Falciparum* parasitaemia and malaria. Statistical significance was set at P < 0.05.

## Results

### Baseline characteristics of the study population

A total of 303 women were enrolled. The mean age of the women was 24.1 ± 5.3 (range: 15–40) years while the mean gestational age was 23.4 ± 5.6 (range: 10–38) weeks. There was a significant positive correlation (r = 0.633; P < 0.001) between maternal age and gravidity. Most of the women attended their first ANC clinic in the second (58.1 %) and third trimester (36.2 %). The majority (68 %) of the women were married. About 90 % of the study participants had at least a primary education and only 11 % had obtained tertiary education. The greater percentage of the women reported a monthly income of less than 30.000 FCFA (franc des Communautés Financières d’Afrique) (approximately 60 USD). Anaemia was frequent (57.3 %) among pregnant women while HIV prevalence of 5.9 % was recorded. ITN usage was 49 % while IPTp-SP uptake was low (11.6 %) (Table 1).

**Table 1:** Baseline characteristics of the study participants

Characteristic (N)	Category	Value (%(n))
Age group (years) (301)	≤20	30.6 (92)
	21–25	32.8 (98)
	>25	36.9 (111)
Gravidity status (300)	Primigravid	28.3 (85)
	Secundigravid	32.7 (98)
	Multigravid	39.0 (117)
Marital status (299)	Single	32.1 (96)
	Married	67.9 (203)
Employment status (298)	Employed	51.0 (152)
	Unemployed	49.0 (146)
Monthly income (USD) (282)	<60	62.4 (176)
	60–100	27.3 (77)
	>100	10.3 (29)
Trimester of first visit (301)	First	5.7 (17)
	Second	58.1 (175)
	Third	36.2 (109)
Type of first clinic visit (300)	Unscheduled	16.0 (48)
	Scheduled	84.0 (252)
Level of Education (295)	Primary	41.0 (121)
	Secondary	47.5 (140)
	Tertiary	11.5 (34)
House surrounding (291)	Vegetation/standing water	39.2 (114)
	Absence	60.8 (177)
Reported fever history (298)	Yes	17.4 (52)
	No	82.6 (246)
Anaemia status (285)	Anaemic	57.2 (163)
	Non-anaemic	42.8 (122)
HIV prevalence (303)	Yes	5.9 (18)
Reported IPTp-SP uptake (299)	Yes	11.6 (35)
	No	87.1 (264)
ITN ownership (296)	Yes	68.9 (204)
	No	31.1 (92)
ITN usage (204)	Yes	48.5 (99)
	No	51.5 (105)

### Prevalence of *P. falciparum* infection and malaria

**Fig. 1:**
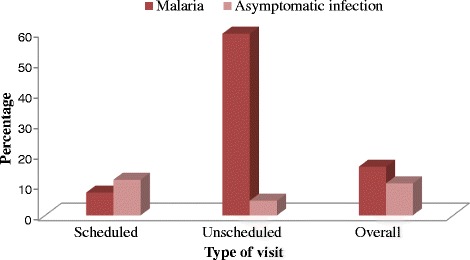
Prevalence of malaria and asymptomatic *P. falciparum* infection among pregnant women at scheduled and unscheduled clinic visits in the Mutengene and Muea Medical Centres

Sixty-eight out of 303 (22.4 %) women enrolled were positive for *P. falciparum* parasiteamia with a GMPD of 529 (range: 30 – 18571) parasites/μl. Geometric mean parasite density differed significantly with marital status, with single women (938) having significantly higher (t = 2.70; P = 0.009) GMPD when compared with married women (338). This difference was independent of age or gravidity status. Temperature was recorded for 257 women, out of whom, 24.9 % (64/257) had febrile illness (temperature >37.5 °C). Malaria parasitaemia was significantly (χ^2^ = 62.34; P < 0.001) associated with febrile status, where a significantly higher percentage of women with febrile illness (64.1 %; 41/64) were parasitaemic for *P. falciparum* infection when compared with aparasitaemic febrile cases (35.9 %; 23/64). The overall prevalence of malaria and asymptomatic infection was 16.0 % (41/257) (95 % CI =11-20 %) and 10.5 % (27/257) (95 % CI = 7.3-15 %) respectively. The prevalence of malaria and asymptomatic infection differed significantly (χ^2^ = 70.7; P < 0.001) with type of clinic visit (Fig. 1). The majority of the malaria cases (61 %; 25/41) were women who reported at the clinic during unscheduled days meanwhile the almost all of the asymptomatic parasitaemic cases (92.8 %; 25/27) were recorded during scheduled visits. There was a significant association between fever and HIV infection. HIV positive women (10.9 %; 7/64) were more likely (OR = 3.3: 95 % CI = 1.1 – 9.7; P = 0.026) to present with fever than no fever (3.6 %; 7/193). However, the majority of (71.4 %; 5/7) of the febrile HIV positive women were co-infected with *P. falciparum*.

### Haematological changes associated with malaria infection

Haemoglobin levels, WBC counts, lymphocyte (counts and percentage) and platelet counts did not differ with the presence of *P. falciparum* parasitaemia or malaria. Nonetheless, a statistical significant negative correlation (r = − 0.34; P = 0.011) was observed between percentage lymphocyte and malaria parasite density. More so, women who presented with fever had a significantly lower (MU = 2.46; P = 0.014) mean percentage lymphocyte (28.63 ± 9.1) when compared with afebrile cases (34.52 ± 13.8).

### Factors associated with of *P. falciparum* infection and malaria in pregnant women

In univariate analysis, maternal age and presence of bush (thick vegetation) and/or standing water near houses were significant factors associated with *P. falciparum* parasitaemia and malaria. Younger women (≤20 years) were frequently infected (P = 0.002) with malaria parasitaemia and commonly had malaria (P = 0.018) when compared with older women (>20 years) (Table 2). Equally, asymptomatic infection was more common in younger women (P = 0.018) than in older women. A significantly higher (P = 0.001) percentage of the women living in houses surrounded by bush and/or standing water had malaria parasitaemia and disease when compared with those with clean house surroundings. Primigravid women frequently (P = 0.043) presented with malaria at first clinic visit when compared with women of older gravidity. IPTp-SP uptake before ANC clinic visit was significantly associated with malaria episode at first visit. Women who had taken SP were likely (P = 0.019) to present with malaria at first visit than those who had not taken SP (Table 2). Out of the ten women who had taken SP and had malaria, seven (70 %) reported for ANC during unscheduled clinic days. Also, women who reported history of fever (26.9 %; 14/52) were more likely (OR = 3.9; 95 % CI = 1.8 – 8.4; P < 0.001) to have taken SP before ANC enrolment compared with those who reported no history of fever (8.6 %; 21/245).

**Table 2:** Factors associated with *P. falciparum* parasitaemia, malaria and asymptomatic infection among pregnant women at first antenatal clinic visit in the study area

Factor	Category	*Pf* parasitaemia		Malaria %(n)	Asymptomatic infection %(n)
		Positive %(n)	Negative %(n)		
Age group (years)	≤20	34.8 (32)	65.2 (60)	25.0 (20)	15.0 (12)
	21–25	14.3 (14)	85.7 (84)	9.0 (7)	9.0 (7)
	>25	19.8 (22)	80.2 (89)	14.1 (14)	8.1 (8)
	χ^2^; P-value	12.17; 0.002		11.96; 0.018	
Gravidity status	Primigravid	30.6 (26)	69.4 (59)	26.8 (19)	9.9 (7)
	Secundigravid	19.4 (19)	80.6 (79)	9.3 (8)	12.8 (11)
	Multigravid	19.7 (23)	80.3 (94)	14.0 (14)	9.0 (9)
	χ^2^; P-value	4.25; 0.12		9.88; 0.043	
Marital status	Single	29.2 (28)	70.8 (68)	20.2 (17)	13.1 (11)
	Married	19.7 (40)	80.3 (163)	14.0 (24)	9.3(16)
	χ^2^; P-value	3.32; 0.068		2.94; 0.23	
Knowledge on MiP	Yes	20.0 (42)	79.5 (167)	12.2 (22)	11.0 (20)
	No	24.7 (21)	75.3 (64)	23.6 (17)	6.9 (5)
	χ^2^; P-value	0.64; 0.425		5.64; 0.06	
Trimester at first visit	First	23.5 (4)	76.5 (13)	26.7 (4)	0 (0)
	Second	21.7 (38)	78.3 (137)	13.6 (21)	11.0 (17)
	Third	23.9 (26)	76.1 (83)	18.2 (16)	11.4 (10)
	χ^2^; P-value	0.19; 0.912		3.37; 0.444	
House surrounding	Vegetation/standing water	31.6 (36)	68.4 (78)	25.0 (24)	11.5 (11)
	Absence	15.3 (27)	84.7 (150)	9.7 (15)	8.4 (13)
	χ^2^; P-value	10.89; 0.001		12.22; 0.002	
IPTp-SP uptake	Yes	28.6 (10)	71.4 (25)	32.3 (10)	0 (0)
	No	22.0 (58)	78.0 (206)	13.7 (31)	11.9 (27)
	χ^2^; P-value	0.77; 0.381		9.74; 0.008	
ITN usage	Yes	21.2 (21)	78.8 (78)	15.1 (13)	9.3 (8)
	No	23.8 (25)	76.2 (80)	17.8 (16)	10.0 (10)
	χ^2^; P-value	0.20; 0.657		0.28; 0.870	

χ^2^ = Pearson chi-square test, Significance level = P < 0.05

MiP = Malaria in pregnancy

*Pf* = *Plasmodium falciparum*

### Risk factors associated with *P. falciparum* infection and malaria in pregnant women

To investigate the risk factors of malaria parasite infection and disease among pregnant women in the study area, multinomial logistic regression model was performed allowing adjustment for possible confounders. Being unmarried (OR = 2.6; P = 0.032) was a significant independent risk factor associated with malaria parasitaemia (Table 3). It is worth noting that the majority of the single women (60.9 %; 56/92) were found in the younger age group (≤20 years) while most of the married women (89 %; 97/109) were in the older age group (>25 years). The difference was statistically significant (χ^2^ = 57.73; P < 0.001). Similarly, there was a significant association (χ^2^ = 55.33; P < 0.001) between marital and gravidity status where the majority of the unmarried women (60 %; 51/85) were primigravidae and the married women predominantly (89.6 %; 108/115) multigravidae. The presence of bush and/or standing water around house surroundings was a significant independent risk factor associated with *P. falciparum* parasitaemia (OR = 3.3; P = 0.002) and malaria (OR = 5.2; P = 0.001) (Table 3). Women with knowledge on MiP were less likely (OR = 0.3; P = 0.037) to present with malaria at first clinic visit (Table 3). Knowledge of MiP vary significantly (χ^2^ = 13.48; P = 0.001) among women of different gravidity status. Multigravid women (43.3 %; 91/210) were more informed about MiP than secundigravidae (34.8 %; 73/210) and primigravidae (21.9 %; 46/210). On the contrary, women with knowledge on MiP (GA = 23.7 ± 5.6 weeks) had their first ANC clinic visit later (t = 2.78; P = 0.006) than those without knowledge on MiP (GA = 21.9 ± 5.1 weeks).

**Table 3:** Risks factors associated with *P. falciparum* parasitaemia and malaria among pregnant women at first antenatal clinic visit in the study area

Factor	Category	*P. falciparum* parasitaemia		Malaria	
		^a^OR (95 % CI)	*P-value	^a^OR (95 % CI)	*P-value
Age group (years)	≤20	1.5 (0.5 – 4.5)	0.50	1.0 (0.2 – 4.0)	0.957
	21–25	0.7 (0.3 – 2.1)	0.575	0.5 (0.1 – 2.0)	0.332
	>25	R		R	
Gravidity status	Primigravid	1.2 (0.4 – 4.0)	0.719	2.8 (0.7 – 11.3)	0.158
	Secundigravid	1.0 (0.4 – 2.7)	0.978	0.7 (0.2 – 2.5)	0.595
	Multigravid	R		R	
Marital status	Single	2.6 (1.1 -6.0)	0.032	1.9 (0.6 – 5.9)	0.254
	Married	R		R	
Trimester at first visit	First	1.8 (0.4 – 8.8)	0.479	2.4 (0.4 – 14.6)	0.331
	Second	0.9 (0.4 – 1.9)	0.717	0.7 (0.3 – 2.0)	0.542
	Third	R		R	
Knowledge on MiP	Yes	0.6 (0.3 – 1.4)	0.244	0.3 (0.1 – 0.9)	0.037
	No	R			
House surrounding	Vegetation/standing water	3.3 (1.6 – 7.0)	0.002	5.2 (2.0 – 14.0)	0.001
	Absence	R		R	
IPTp-SP uptake	Yes	2.0 (0.7 – 5.7)	0.222	4.6 (1.3 – 16.4)	0.019
	No	R		R	
ITN usage	Yes	1.1 (0.5 – 2.2)	0.862	1.2 (0.4 – 3.0)	0.785
	χ^2^; P-value	R			

R = Redundant

*Significance level obtained by multinomial logistic regression analysis

^a^Odd ratio adjusted for all possible confounders

## Discussion

It is generally assumed that due to acquisition of partial immunity to malaria, parasitaemic women living in areas of stable transmission do not often present with symptoms [2]. However, the present study demonstrates that, among pregnant women reporting for first clinic visit, malaria (16.0 %) was prevalent when compared with asymptomatic parasitaemic cases (10.5 %) and women with malaria frequently report for medical attention during unscheduled clinic days. Hnynh et al. [5] in Benin have reported similar findings. Malaria-associated acute symptoms such as fever will likely cause pregnant women to seek medical care during unscheduled days [5]. Comparable to previous reports in Cameroon by Leke et al. [9] (7.9 %) and Mbu et al. [10] (6.6 %) malaria cases were less common (7.5 %) among women reporting for ANC during routine clinic visits. Certainly, women who are apparently well (afebrile) will generally report for first ANC during scheduled visit days as observed in the present study. Women reporting at the clinic during unscheduled visits represent a high risk group and so prompt malaria diagnosis and proper treatment to avoid adverse consequences of early *P. falciparum* infection in pregnancy is justified [6, 8].

Haematological changes in malaria, such as thrombocytopenia and leucocytosis or leucopenia are well-recognized [13–15]. Haemoglobin levels, WBC and platelet counts did not discriminate malaria from asymptomatic parasitaemia. This might be partly because most of the women with symptomatic infection were mild malaria cases. Significant morphological and numerical changes in all the blood cell lines observed in malaria are usually dependent on the disease severity (complicated versus uncomplicated malaria) [25] among other factors such as *Plasmodium* species [26, 27] and the immune status of an individual [28]. In a Ugandan study, *P. falciparum* uncomplicated malaria did not produce significant changes in the total WBC count, differential WBC count and RBC indices. The authors suggested that haematological changes are unreliable laboratory indicators of malaria in acute uncomplicated *Plasmodium falciparum* malaria [29]. Nevertheless, the level of malaria parasitaemia was associated with lymphocyte percentage. Lymphopenia which is sometimes profound but transient or temporary, is a common finding in acute malaria in non-immune adults [25, 30] as well as in children living in malaria endemic areas [13, 25]. The lymphocyte percentage being inversely proportional to the level of parasitemia reflects redistribution of lymphocytes (due to extravasation and homing either at inflammatory sites or at secondary lymphoid organs (e.g. peripheral lymph nodes and spleen)) or lymphocyte destruction due to Fas-induced apoptosis [31]. Lymphopenia is a classical feature in arthropod-borne viral infections [32]. Arboviruses can both cause lymphopenia and be associated with increase level of malarial parasitaemia. Co-infection of malaria and other febrile illness caused by arthropod-borne infections such as dengue has been reported [32].

This study investigated the risk factors for *P. falciparum* parasitaemia and malaria in pregnant women. The findings show that household proximity to bush and/or standing water increased risk of *P. falciparum* parasitaemia and malaria in the pregnant women residing in such areas. Similarly, previous findings from a study carried out in this area by Akenji et al. [33] revealed a positive significant association between state of house surrounding and risk of malaria parasite infection. Bushes and/or standing water serve as breeding sites for the malaria parasite vectors. Wanji et al. characterised anopheles breeding sites in the mount Cameroon area during peak to late rainy season period (August-November) [34]. The study showed that about 94 % of the breeding sites in this area are temporary water bodies, of which about 81 % are productive breeding sites (contained *Anopheles* larvae). The majority (86 %) of these breeding sites are found within 20 m from the nearest inhabited house while 21 % are located between 20 and 50 m from the nearest inhabited houses. Breeding sites are less likely to occur beyond a distance of 50 m. Furthermore, Tanga et al. [19] reported a high parous (an indication of haematophagy) rate (≤70 %) for all anopheline species further suggesting availability of potential breeding sites close to human residence.

In malaria endemic settings, studies have consistently shown that primigravid women are at increased risk of malaria [3, 4, 6, 10]. In conformity with previous findings, primgravidity was a significant factor associated with malaria. Greater risk of infection and adverse outcome in primigravidae has been attributed to the absence of pregnancy- specific malaria immunity to pregnancy-associated variant surface antigens (VSAPAM) that predisposes them to malaria severity [35]. The fact that human residence in areas with nearby bushes and/or standing water was an independent predictor of *P. falciparum* infection and malaria suggests that the environment may modify the risk of infection and disease in pregnant women in this setting rather than gravidity. Areas of intense malaria transmission risk have been associated with greater risk of infection [11]. The association observed between knowledge of MiP and malaria is not possibly a cause-effect relationship. Rather, it can be explained by the fact that the majority of the women who had some knowledge of MiP were multigravidae. Multigravid women are less likely to be susceptible to malaria in pregnancy due to acquired gravidity-dependent pregnancy associated immunity [35]. Women of older gravidity become enlightened about MiP either through past ANC clinic attendance or experiences. Reported IPTp-SP uptake was low. This is because the study population involved women reporting for their first clinic visit and about 90 % coverage of at least one SP dose is achieved after first ANC consultation in the study area [17]. SP uptake correlated with malaria at ANC enrolment. In peri-urban and rural communities, pregnant women with malaria often prefer to self medicate through drug store and herbs. Generally, these women seek treatment in health facilities as a last resort, usually when the disease poses a major threat to life [36]. SP is an inappropriate treatment for malaria [8] and misuse of SP may have implications on resistance against SP for malaria prevention in pregnancy. There is urgent need to evaluate SP efficacy as IPTp in this setting and to encourage pregnant women to seek appropriate diagnosis and treatment of febrile illness at the medical centre.

In this study, more than twice (OR = 2.6) as many unmarried pregnant women were infected compared with women with a husband or partner. More so, single women had higher parasite densities than did married women. Similarly, in Yaoundé, Mbu et al. [10] showed that single women had a 4-fold increase risk of developing malaria during pregnancy. Increased risk of *P. falciparum* among single women might be due to the fact they were significantly younger (≤20 years) and the majority were primigravidae. In areas of high and stable transmission, age and pregnancy-associated anti-parasite immunity may play an important role in limiting *P. falciparum* infection to low parasite densities [35, 37] in older and multigravid women respectively. Anchang-Kimbi et al. [16] reported higher prevalence of low-grade parasitaemia in older women (>20 years) than younger women (≤20 years) in the study area. The fact that single women had higher parasite densities when compared with married women irrespective of age and gravidity status suggests that other factors may account for high parasite load in single women. It will be interesting to investigate the role of marital status in the risk of malaria parasite infection in this study area. Numerous studies have shown advantages of being married on the health of individuals [38]. Marriage could improve health outcomes in a variety of ways: It may improve economic well-being [39], health outcomes by enhancing access to health care or lowering stress. In addition, a spouse may play an important role in monitoring and encouraging healthy behaviours [40]. Chepkemoi and Mutulei [41] suggested that partner support is critical for effective prevention of malaria in pregnancy. The support of a husband or partner may play a key role on the woman’s timing of her first ANC. Some studies show that women not supported by a partner are likely to report for late antenatal care enrolment [42]. This in turn could delay early uptake of IPTp-SP.

This study had few limitations. First, the study did not investigate other arthropod- borne infections that cause fever. In Africa, fever episodes are most often considered as malaria (without any laboratory confirmation) and some of the supposed malaria attacks could be actual dengue fever which remains largely unknown [43]. Two serotypes; dengue virus (DENV) -1 and DENV-2 have been, or are currently, circulating in Cameroon [43, 44] although unrecognized by individuals and even by medical personnel [43]. In Cameroon, the vast majority of DENV infections are unapparent or oligosymptomatic and this form of presentation of the disease may under represent its distribution and impact [43]. In addition, Fokam et al. [44] reported anti-viral antibodies against some members of *Alphavirus* (Chikungunya and O’nyong-nyong) in sera of patients from Mt Cameroon area. Secondly, the study did not survey the environment where people stay to ensure that the reported presence of standing water and bushes around human residence faithfully relate to the risk of *P. falciparum* and malaria. While anopheline mosquitoes are predominant (82.73 %) and diverse in the Mt Cameroon area, Tanga et al. [19] identified *Aedes*, *Culex* and *Mansonia* species in some mosquito breeding sites and thus the possibility of DENV transmission in the area cannot be rule out. It is imperative that future studies investigate the prevalence of arboviral infections among pregnant women in this study area, as these infections are important and overlooked public health problems.

## Conclusion

In the study area, *P. falciparum* parasitaemia is a leading cause of febrile illness among pregnant women before first ANC clinic visit and women with malaria frequently seek for medical attention during unscheduled clinic days. Being unmarried and the presence of bush and/or standing water in house surroundings modify the risk of *P. falciparum* infection and malaria in pregnancy. Prompt malaria diagnosis and proper treatment as well as early ANC care attendance to avoid adverse consequences of early infection in pregnancy is justified. Education on early ANC attendance and environmental sanitation are important public health targets for malaria control in pregnancy in this setting.

## Abbreviations

ANC: Antenatal care clinic
IPTp-SP: Intermittent preventive treatment during pregnancy with sulphadoxine-pyrimethamine
ITN: Insecticide-treated nets
WBC: White blood cell
